# Three-dimensional morphological changes of potato slices during the drying process

**DOI:** 10.1016/j.crfs.2021.11.009

**Published:** 2021-11-27

**Authors:** Li Sun, Pengqi Zhang, Xin Zheng, Jianrong Cai, Junwen Bai

**Affiliations:** a School of Food and Biological Engineering, Jiangsu University, Zhenjiang, Jiangsu, 212013, PR China; b School of Mechanical Engineering, Jiangsu University, Zhenjiang, Jiangsu, 212013, PR China

**Keywords:** Potato drying technology, Three-dimensional models, Sensor technology, Morphological changes, Rate of change in the mean height

## Abstract

Hot air drying is a common method for drying potato slices. In this paper, the three-dimensional (3D) morphological changes incurred during hot air drying of potato slices were investigated. The effect of drying on the thickness and diameter of potato slices was of special interest. The results showed that the potato slices underwent stages of regular warping, collapse, and curling during the drying process. After classifying the numerical variation in characteristics into the standard deviation in mean height (SDMH) and the rate of change in mean height (RCMH) of potato slices, the RCMH was selected to describe the 3D morphological changes in the potato drying process. A critical point and a termination point for RCMH of potato slices in the drying process were observed. Samples varied widely after the critical point was reached. A logarithmic function model was used to assess differences in the RCMH at the critical point and the termination point. The R-squared (R2) value of 0.9 suggested a strong correlation between the parameters of the experiment and changes in slice thickness and diameter. The model proposed in this paper could accurately characterize the late-stage changes in potato slice qualities during hot air drying of potato slices.

## Highlights

• Drying potato slices underwent stages of regular warping, collapse and curling.
• A correlation between the fitted results and the changes in thickness and diameter.
• A height change model in the later stage of hot air drying of potato slices.

## Graphical abstract

**Figure:**
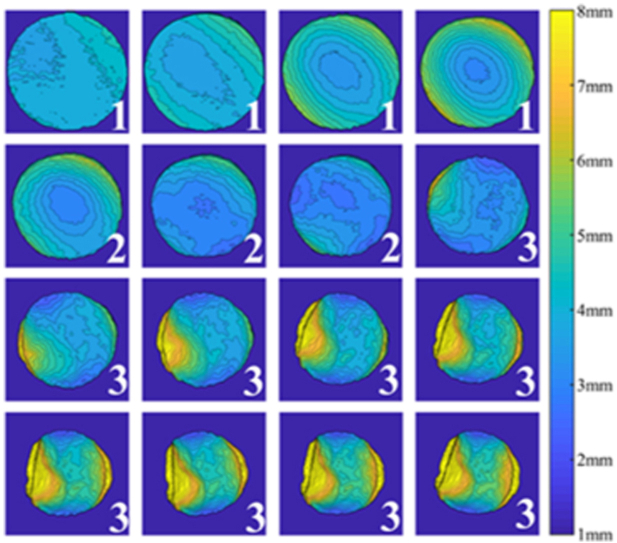

## Nomenclature

*SD*: The standard deviation
*SDMH*: The standard deviation in mean height
*RC*: The rate of change
*RCMH*: The rate of change in mean height
*i*: A Horizontal coordinate value
*j*: A Vertical coordinate value
*G(i, j)*: The grayscale image pixel position at (i, j)
*H(i, j)*: The height value position at (i, j)
*H*: The mean height value in the potato slice image
*H(p*_*i*_*)*: The height value of p point
*H*_*t*_: The mean height value in the potato slices at time t
*H*_*0*_: The mean height value in the potato slice image before drying
*M*: The Collection of point p.
*m*: The number of points in the set M

## 1 Introduction

The potato is the fourth most important food crop in the world, after wheat, rice, and corn (Luo et al., 2015). Potatoes are primarily composed of starch and also contain small amounts of protein, minerals, vitamins, and fiber (Graciela et al., 2020, Huang et al., 2018). As potatoes contain the eight essential amino acids that the body cannot synthesize, they are especially nutritious. Unlike other food crops, potatoes have a very comprehensive vitamin content. Hence, even a small number of potatoes can meet a person's daily vitamin consumption (Wang et al., 2019). In China, potatoes are defined as a staple food by the Ministry of Agriculture. In 2020, 17 million tons of potatoes were grown over 475,807 hectares of land. As a seasonally harvested crop with high water content, potatoes are prone to greening, sprouting, and rotting during storage (Huang et al., 2017). The resulting increases in solanine content can cause food poisoning, and associated symptoms such as nausea, vomiting, abdominal pain, dizziness, and respiratory distress (Cheng et al., 2020). Drying is an important method to prolong the shelf life of potatoes. By significantly reducing the water content of potatoes, drying inhibits internal microbial activity and reduces storage losses of potatoes (Xi et al., 2018).

Of the many methods of drying potatoes, hot air drying is the most common. As potato tubers are 80% water by weight, they shrink during drying. Water loss from porous and hygroscopic potatoes causes them to deform and crack during shrinkage, which greatly affects the quality of the finished product after drying (Mahiuddin et al., 2018, Oikonomopoulou et al., 2011, Yadollahinia and Mehdi, 2009). Many factors affect the drying results, and different drying conditions may result in different qualities. Hence, changes in potato slices during hot air drying warrant a thorough investigation to inform researchers about the potential benefits of the hot air drying process of potatoes.

Current research in the hot air-drying process mainly focuses on detecting one or more parameters such as color, shrinkage, moisture content, and drying time (Zhu et al., 2021). For example, Guo et al. (2020) mainly studied the effect of different hot air-drying temperatures on quality parameters such as color difference and browning degree of hawthorn hot air-dried products. They showed that drying temperature positively correlated with the degree of browning, redness, and color difference, but negatively correlated with the brightness (Guo et al., 2020). Using online monitoring devices, Nadian et al. (2016) monitored how the attributes of kiwifruit slices, such as color and shrinkage, change during hot air drying (Nadian et al., 2016). Both abovementioned studies mainly focused on color detection of two-dimensional (2D) images. Wijitha et al. (2020) used four empirical mathematical models, to investigate the effect of different hot air-drying temperatures on the shrinkage of persimmon slices during hot air drying. Their experiments showed that the drying temperature influenced the rate of shrinkage and that a quadratic term model could provide the best characterization of persimmon volume shrinkage with moisture content (Senadeera et al., 2020). Weiqiao et al. (2020) developed nuclear magnetic resonance/imaging and microwave vacuum drying equipment to measure and quantify water content and moisture distribution of foods undergoing drying (Lv et al., 2018). Zhang et al. (2012) designed a wireless real-time monitoring system to detect online detection of water content during the freeze-drying process of fruits and vegetables (Zhang et al., 2016). Li et al. (2019) designed a device to measure the moisture content of fruits and vegetables undergoing hot air-drying. This device displayed intelligent control of hot-air drying of fruits and vegetables (Li et al., 2019). While these recent papers mainly focus on the change of moisture and its effect, which rely on the essence of drying, analyses measuring the effect of external parameters are lacking.

Studies on the morphological aspects of drying have focused on 2D projection characteristics and shrinkage properties. For instance, changes in characteristics of 2D projections of potato slices and other agricultural products during drying have been studied (Yadollahinia and Mehdi, 2009, Yan et al., 2008). Similarly, the drying shrinkage characteristics of food materials under different drying conditions have also been examined (Shekofteh et al., 2012, Singh, 2020). Other studies have studied the effects of drying on morphological change by simulation. Most recently, Raimundo et al. (2020) used shrinkage lumped models to study the effect of different drying conditions on banana fruit (Pereira de Farias et al., 2020). Previous dry research focused on 2D-based data, which is less accurate than the more newly developed three-dimensional (3D) information acquisition technology. With the advent of 3D sensor technology, 3D information research has been heavily used in agriculture, including its use to reconstruct plant leaves and orchard picking operations (George et al., 2013, Sepehr et al., 2019). Additionally, material drying research tends to be combined with 3D information research as the added dimension conveys more effective information. Therefore, this study mainly examined morphological changes in 3D during the drying process of potato slices.

This study analyzed the mean height change pattern of potato slices during the drying process in 3D. Two parameters, the standard deviation in mean height (SDMH) and the rate of change in mean height (RCMH), were examined. The SDMH characterized the variation of height and the RCMH characterized the curling speed during the drying of potato slices. This study analyzed the effect of thickness and diameter on the above two parameters, as a reference for detecting the quality change of potato slices during the drying process.

## 2 Materials and methods

### 2.1 Samples

Twenty fresh potatoes were purchased from the Kaiyuan supermarket in Zhenjiang P.R. China. Potatoes that were at least 5 cm long were placed in an indoor environment at 17°C and 30% relative humidity (RH) for one day. Before the experiment, the potatoes were washed to remove the soil from the skin. A household hand-cranked fruit and vegetable slicer were used to slice potatoes into three different thickness: 2 mm, 4 mm, 6 mm. A ring cutter was used to cut the center of each potato slice into round slices of the required diameter: 22 mm, 33 mm, 44 mm. At any given time-point, the height of each slice was measured by a Gocator 3210 3D sensor. The mean height of experimental samples fluctuated in a small range (2 ± 0.2 mm, 4 ± 0.2 mm, 6 ± 0.2 mm).

### 2.2 Experiment equipment

#### 2.2.1 Homemade tubular hot air dryer

**Fig. 1:**
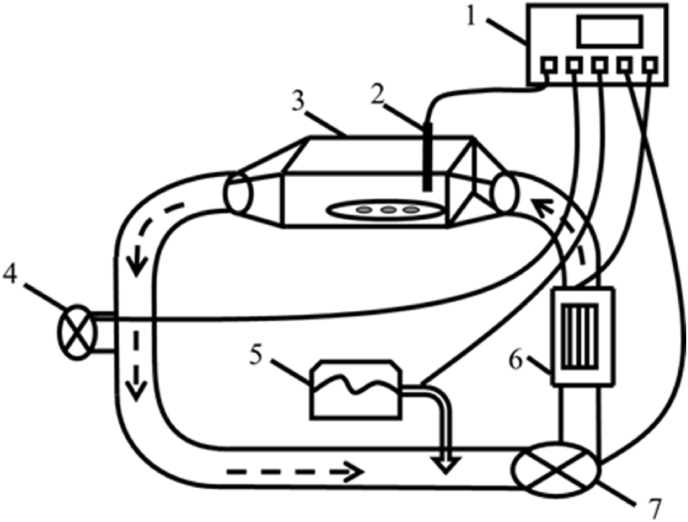
**Structure of the homemade tubular hot air dryer.** The components include a 1. Control box; 2. Temperature and humidity sensor; 3. Drying room; 4. Dehumidifier fan; 5. Steam humidifier; 6. Heating device; 7. Centrifugal fan.

A homemade tubular hot air dryer (Fig. 1) was used to dry the 6 potato slices that were prepared before the experiment.

The drying chamber was equipped with a temperature and humidity sensor (Fig. 1). The temperature sensor could detect a wide range of temperatures (−40 to 120°C) with ±0.5°C accuracy. The humidity sensor could detect a full range of humidity (from 0 to 100% RH) with a ±5% RH accuracy. After receiving data from the temperature and humidity sensors, the control box adjusted the temperature, humidity, and wind speed by modulating the drying room, dehumidifier fan, steam humidifier, heating device, and centrifugal fan. The control mode of temperature and humidity The Proportional Integral Derivative (PID) control mode could detect a temperature range of -40 - 110°C with ±1 °C accuracy. The humidity range of 0–100% RH was detected with ±3% accuracy. The wind speed control, operated by an open-loop control, could produce wind speeds up to 13 m s ^-1^.

#### 2.2.2 3D information detection platform

**Fig. 2:**
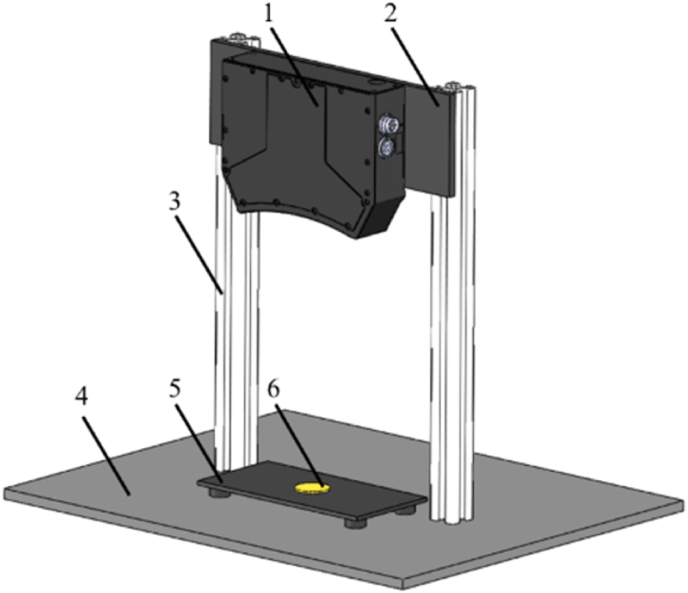
**The 3D information detection platform.** The platform was comprised of a 1. Gocator 3210 3D sensor; 2. Sensor fixing plate; 3. Aluminum materials (3030); 4. Base plate; 5. Material pallet; 6. Potato slice.

A Gocator3210 3D point cloud detection platform was developed (Fig. 2). To detect potato slices above the material pallet, a sensor fixing plate and aluminum materials (3030) were used to fix the Gocator 3210 3D sensor to the base plate. The optical pattern was projected onto the test object. The Gocator 3210 structured light sensor decoded the depth information contained on the object surface. The sensor generated 3D point cloud data as a set of vectors in a 3D coordinate system. This sensor solved the light-scattering problem of light which would otherwise be detrimental when scanning a shiny or curved surface. The sensor measurements range was as follows: 71.0 × 98.0 - 100.0 × 154.0 mm^2^ fields of view, 0.060–0.090 mm resolution, and within an accuracy of ±0.035 mm.

To obtain stable and reliable 3D point cloud data, the platform was placed on a smooth and vibration-free table and the sensor was mounted at the center of the field of view 220.9 mm above the base plate. The test sample was placed on a material pallet directly below the camera. The 13 mm tall material pallet brought the total mounting height of the camera up to 233.9 mm. After 5 measurements, the actual total mounting height of the camera was determined to be 236.44 mm.

### 2.3 Experimental design

Samples were dried at 70°C, 4 m s ^-1^ wind speeds, and 10% humidity. The diameters of potato slices were 22 mm, 33 mm, and 44 mm, and the thicknesses were 2 mm, 4 mm, and 6 mm, respectively. This two-factor experiment consisted of three levels and a total of 9 groups of experiments. Each experiment group was repeated three times was consistent results.

The following experimental procedure was followed: *First*, washed potatoes were peeled and cut into slices using a slicer. *Second*, the equipment was turned on and the system parameters were set. The drying environment was stabilized after the hot air dryer ran for 34 min. *Third*, of the six potato slices used in each group, four potato slices were used to obtain 3D parameters and two potato slices were used for weighing. 3D data and weight acquisition were performed on the potato slices every 5 min. *Finally*, when the rate of weight loss of potato slices was less than 0.01 g, the drying was terminated and the hot air dryer was turned off.

### 2.4 Data processing

#### 2.4.1 3D information conversion

**Fig. 3:**
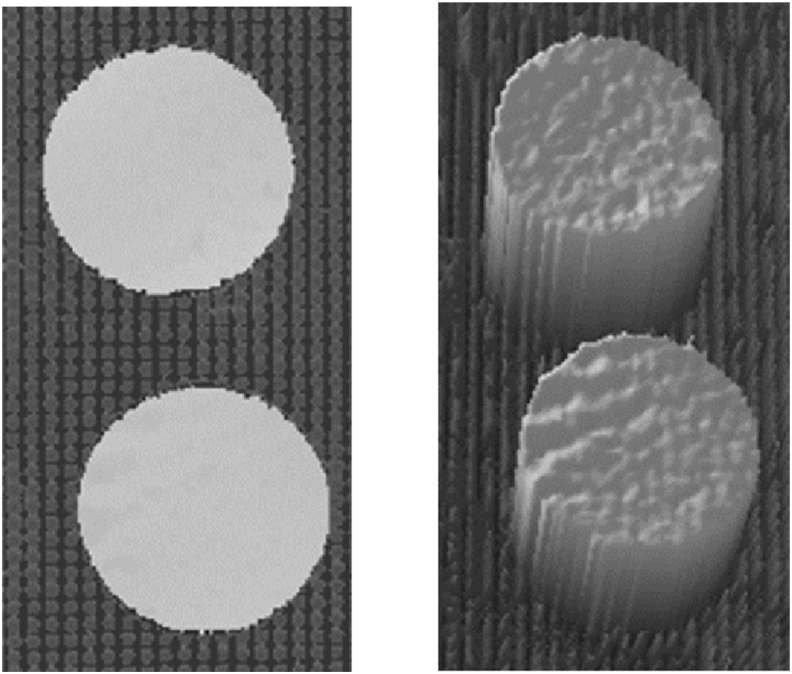
**Sample data generated after altering 3D point cloud data.** Grayscale image (left) and 3D map (right) of potato slices.

3D information obtained from the platform was saved as 3D point cloud data. The large data was converted to 2.5D grayscale images to avoid poor real-time 3D point cloud data processing. Using this method, grayscale images and corresponding 3D maps were generated (Fig. 3).

As the potato slices on the Teflon fleece were significantly taller than the background, they could be easily distinguished (Fig. 3). Water on the surface of the potato slices caused the camera to fail to match stereoscopically. Holes were created on the surface to circumvent this problem. Mixed pixels at the edge area of the potato slices indicate mismatches at the transition zone between the edge of the potato slice and the background.

#### 2.4.2 Grayscale image processing

**Fig. 4:**
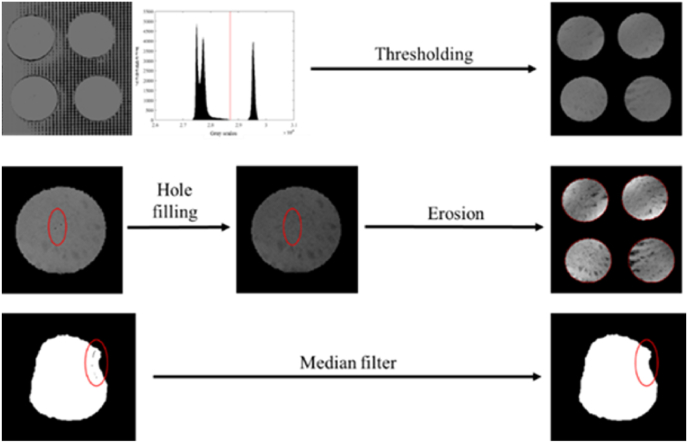
**Image processing flow.** After thresholding and hole-filling, erosion was adjusted, and the principle of the median filter was applied.

Image processing mainly involved thresholding, hole filling, erosion, and median filter (Fig. 4). Thresholding involved accurately extracting the potato slice region. The grayscale histogram analysis of the image showed that the grayscale value distribution of the background and potato slices on the image produced two obvious peak profiles. Therefore, we used the two peaks thresholding method. As 27400 was the lowest point on the valley between two peaks, it is selected as for image thresholding based on the following equation:

(1) $$G\left(i,j\right)=\left\{ \begin{array}{l} G\left(i,j\right)\;\;\;if\;\;\;G\left(i,j\right)>27400\;\;\\ 0\;\;\;\;\;\;\;\;\;\;\;if\;\;\;\;G\left(i,j\right)<27400 \end{array}\right.$$

where *G*(*i*,*j*) is the grayscale image pixel position at *i*, *j*.

To fill points excluded by binocular stereo-vision matching, the average gray values of the 7 × 7 pixels in the neighboring windows were replaced with gray values of all pixels outside the specified gray value range (27400–32767).

Erosion removed mixed pixels at the edge of potato slices. A circular structure of a 3.5-pixel radius was used to correct for erosion and effectively remove mixed pixels from the edges of the potato slice.

The principle of the median filter was used to replace the value of a point in a digital image with the median of the values of the points in a neighborhood of that point. This correction ensured that the surrounding pixel values were close to the true value and eliminated isolated noise points. Median filter used 2 × 2 pixel rectangular windows to repair large areas of holes caused by excessive curling.

#### 2.4.3 Height data extract from grayscale images

After image-processing, the data was extracted as 16-bit grayscale images with an accuracy of 0.003 mm. Standard planes of six different thicknesses were placed on the 3D information detection platform to obtain 3D point cloud data. The mean grayscale values corresponding to planes of different heights were calculated (Table 1).

**Table 1:** The heights of the six standard planes and the corresponding mean grayscale values.

	No.1	No.2	No.3	No.4	No.5	No.6
Plane height (mm)	0.00	4.79	16.86	29.04	39.51	63.95
Mean grayscale	27136.8	28734.0	32757.0	36818.6	40307.7	48455.1

**Fig. 5:**
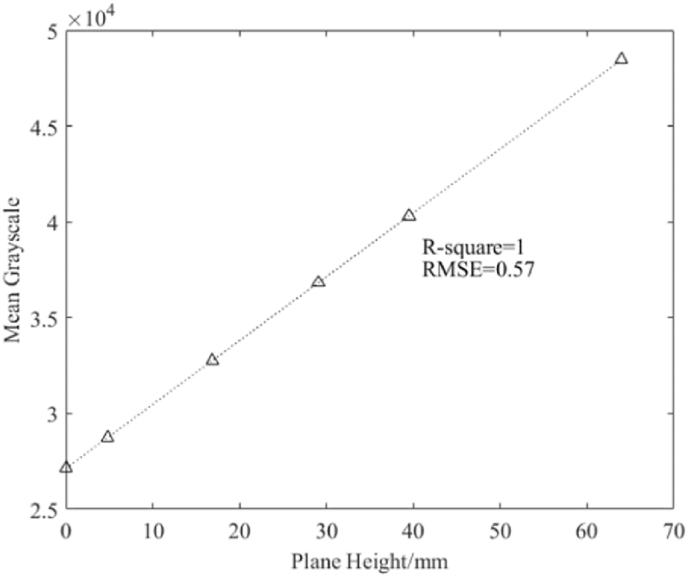
**A linear relationship between plane height and mean grayscale values.** The original data points and the corresponding function.

The analyses revealed a linear relationship between plane height and mean grayscale. This linear fit of the original data (Table 1) was also captured graphically (Fig. 5).

The function model obtained from the fit is shown in Equation (2).

(2) G(i,j)=333.3×H(i,j)+27140

where *H*(*i*, *j*) denotes the height at *i*, *j* in the array of the plane height.

#### 2.4.4 Calculation of the SDMH and RCMH

The mean height represented the degree of curling of the potato and closely correlated to the morphology. However, as there were differences in the mean height between each group, the standard deviation in mean height (SDMH) and the rate of change in mean height (RCMH) values extracted from the height means were used instead.

The SDMH characterized the variation of height and the RCMH characterized the curling speed during the drying process. The analysis of SDMH and RCMH was used to explore the temporal variation of potato slices in the vertical direction during the drying process.

The calibration of the 3D camera ensured that the height data obtained from the experiment was accurate. The SDMH and RCMH values were calculated as shown in Equation (3).

(3) $$H=\frac{\sum_{p_{i}\in M}H\left(p_{i}\right)}{m}\;SD=\sqrt{\frac{\sum_{p_{i}\in M}{\left(H\left(p_{i}\right)-H\right)}^{2}}{m-1}}\;RC=\frac{H_{t}-H_{0}}{H_{0}}\times 100\%$$

where *H* denotes the mean height value in the potato slice image, *H*(*p*_*i*_) denotes the height value of *p* points, and *m* denotes the number of points in the set *M*. Here, *SD* refers to SDMH and *RC* refers to RCMH. *H*_*t*_ denotes the mean height value in the potato slices at time *t* while *H*_*0*_ denotes the mean height value in the potato slice image before drying.

## 3 Results and discussion

### 3.1 Time-varying analysis of drying process

**Fig. 6:**
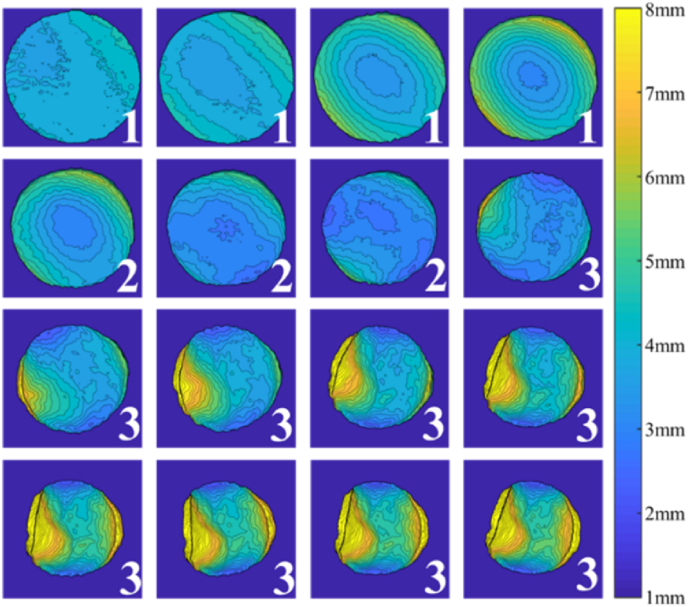
**Time-varying contour map of a potato slice height.** The three stages of drying were represented in pseudo-color. A scale (1–8 mm) is provided as a reference for assessing changes in height. (For interpretation of the references to color in this figure legend, the reader is referred to the Web version of this article.)

The observed trend of change during drying of potato slices of different thicknesses and diameters was similar. A representative example of the time-varying condition of potato slices during the drying process is provided for data visualization purposes (Fig. 6). This potato slice had a thickness of 3 mm and a diameter of 33 mm and was dried by hot air for 75 min. Images were captured every 5 min.

Analyses of the projection of the potato slices revealed that both the projected area and the roundness were gradually shrinking throughout the drying process. As the slices were dehydrating, an imbalance of internal and external pressure caused them to shrink. Additionally, the hot drying air caused potato slices to rotate and change location (Fig. 6). This accounted for warped potato slices that formed at different times in different positions. Analyses of the contour distribution of potato slices could also provide insights on the change in height of potato slices that have gone through the three stages of drying (Fig. 6).

During the initial drying stage (Label 1, from 0 min to 15 min), the temperature of the inner ring was lower than that of the outer ring (Yang et al., 2001). This uneven temperature field caused inconsistent water loss at different locations during the drying process and led to the warping of potato slices. As the outer layer was warmer, the height of the inner ring was lower and increased more gradually. Not only was the outer ring warmer, but it was also curly and the height was greater than the thickness of the cross-section. Collectively, this suggested that the slice height gradually increases outward from the inner ring.

During the intermediate stage of the drying process (Label 2, from 20 min to 30 min), the moisture in the material capillary and the solid skeleton began to migrate outward, and the thermal stress initially increased before decreasing as the moisture ratio decreased (Alireza et al., 2009). When the internal thermal stress exceeded the ultimate pressure of the cell wall, the microporous structure began to collapse (Joardder et al., 2015). During this stage, the overall height of the potato slices dropped below the initial thickness. Differences in the material account for irregularities in the height distribution. Potato slices at this stage tended to be at an even temperature with only subtle curls.

In the final stage of the drying process (Label 3, from 35 min to 75 min), the thermal stress gradually decreased and the water in the cells gradually migrated out. The solid yet elastic skeleton of potato slices gradually became more glassy (Joardder et al., 2015). The potato slices appeared hardened and curled, with height levels that continued to increase. By 55 min, the skeleton of the potato slices assumed their final shape, after which point, the height remains unchanged and the curling was more apparent.

**Fig. 7:**
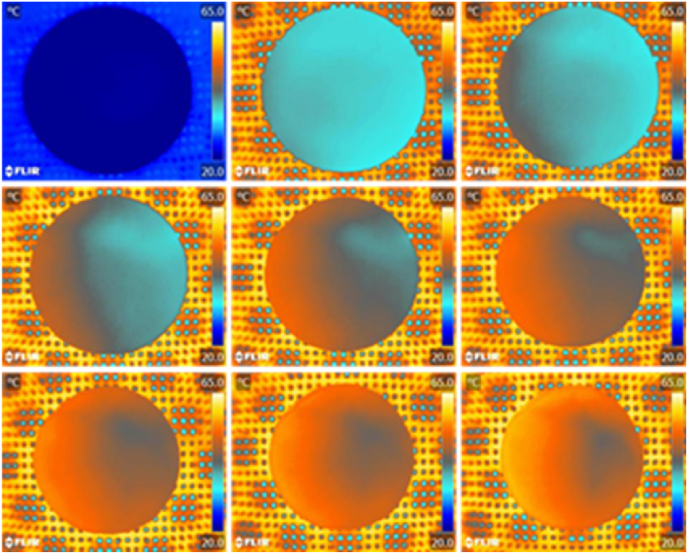
**Time-varying thermal imaging of a potato slice height.** FLIR A35 infrared thermal imaging camera was used to capture images over 40 min.

To validate the occurrence of the three stages observed above, the same sample was captured by FLIR A35 infrared thermal imaging camera for 40 min at a 5-min sampling interval. Using this approach, nine infrared thermographic images were acquired (Fig. 7).

Hot air blown from the left caused the left side of the potato slice to warm up faster (Fig. 7). During the initial stage (from 0 to 15 min), heat gradually transferred from left to right, especially from the periphery to the center. In the intermediate stage (from 20 to 30 min), the temperature was more even throughout the sample. Beyond this stage, the peripheral temperature continued to rise and heat continued to be transferred to the center. Additionally, since the temperature increased faster on the left side, the left side also experienced the intermediate stage for a shorter duration. This might have resulted in the main curl starting from the right side.

### 3.2 Time-varying analysis for the SDMH

**Fig. 8:**
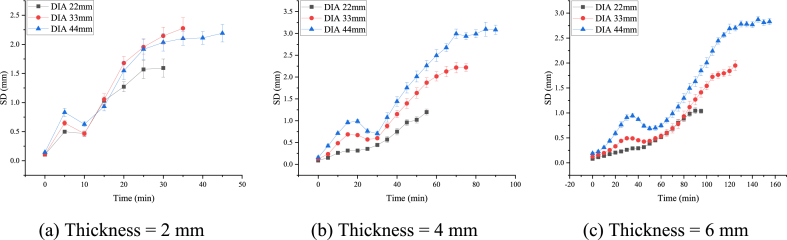
**The SDMH value change over time.** Regardless of thickness, SD increased throughout the drying process.

The SDMH value of potato slices was captured over the course of the drying process (Fig. 8). In general, there were large increases in the SDMH of potato slices during the drying process.

During the early stages of drying, the SDMH values of all potato slices were close to zero. Since potato slices have an inherent slight curl after slicing, none of the SDMH values began at zero. As the potato slices started drying, some slices displayed a modest decrease in SDMH values as they were in the collapse stage. Potato slices of larger diameter were more likely to display the decreased SDMH value during the collapse stage.

Additionally, the SCMH at the termination point for potato slices with a thickness of 2 mm and a diameter of 44 mm was less than that observed in potato slices with a thickness of 2 mm and a diameter of 33 mm. This phenomenon only occurred in potato slices of 2 mm thickness. Further analysis revealed that the small thickness (2 mm) of the potato slices led to structures that cannot curl during the later curling process.

This phenomenon was not easy to quantify because although the SDMH values started at similar inconsistent points, not all groups of potato slices show a decrease in SDMH values during the collapse stage. Hence, the use of SDMH values to describe the 3D morphological changes in potato slices during drying was not representative of all slices.

### 3.3 Time-varying analysis for the RCMH

**Fig. 9:**
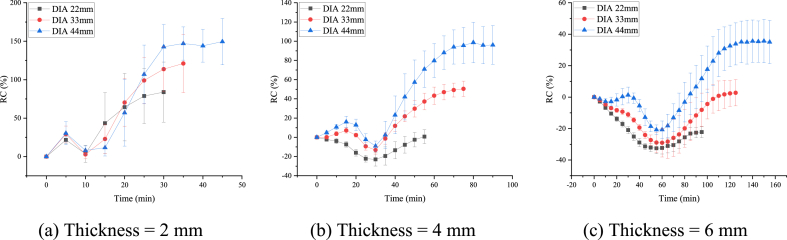
**The RCMH value change over time.** RCMH increased over the course of the drying process.

The RCMH value of potato slices in the drying process revealed a short phase of decrease followed by a large increase in the RCMH of potato slices during the drying process (Fig. 9).

Although surface water and intercellular water in potato slices evaporated during the early stages of drying, it had a minimal effect on the contraction of the material. Therefore, the RCMH of individual potato slices was more consistent, and the differing diameters did not exert a noticeable effect. Thinner potato slices experienced more warping, which was especially obvious at this stage. The difference in RCMH of 2 mm thick potato slices with different diameters was not significantly different from 4 mm to 6 mm thick slices (Fig. 9).

In the late drying stage, the potato slices changed from their mobile elastic state to a less mobile and highly viscous glassy state. This change was accompanied by rapid increases in the RCMH. Potato slices are complex in composition and structure that showed a great deal of variability between individuals undergoing the drying process. Generally, slices with larger diameters also had larger final average heights.

After drying proceeded for some time, the cell membrane of the potato slices collapsed and the water inside the cells gradually migrated outward. This resulted in an increase in the RCMH from its previous downhill trend (Halder et al., 2011). This turning point was referred to as the ‘critical point’.

Based on the time-varying properties of the RCMH, we selected the RCMH at the critical and termination points as indicators of air drying.

The RCMH at the critical and termination points and their associated standard deviations (SD) for potato slices of different thicknesses and diameters during the drying process were summarized (Table 2). The RCMH at the critical point decreased as thickness increased of slices with the same diameter but different thicknesses. The same trend was observed for the termination point. When the thickness was the same but the diameters varied, the RCMH of the termination point increased with an increase in diameter. And at the critical point, the absolute value of the RCMH decreased as diameter increased, except for the group with 2 mm thick slices with 44 mm diameters.

**Table 2:** The rate of change of the average height at the critical and termination points.

Thickness (mm)	Diameter (mm)	Critical Point		Termination Point	
		RC(%) a	SD (%) b	RC(%) a	SD(%) b
2	22	3.67	11.01	83.86	39.03
	33	2.92	5.66	121.10	37.83
	44	8.00	5.87	149.42	30.17
4	22	−22.98	6.92	0.64	7.15
	33	−13.27	4.15	50.48	7.90
	44	−9.09	5.19	96.06	20.06
6	22	−32.51	3.45	−22.21	3.55
	33	−29.02	9.04	2.75	8.33
	44	−20.78	3.11	35.03	13.68

a The rate of change.

b Standard deviation.

All nine groups of different thicknesses and diameters showed the same pattern of the RCMH at the termination point being higher than the critical point. As mentioned previously, the group with a thickness of 2 mm and a diameter of 44 mm at the critical point did not quite fit the above pattern. When thickness was 2 mm, the mean value of the height at the critical point increased by 0.16 mm (an 8% increase) from the initial value.

### 3.4 Analysis of the change from the critical point to the termination point

The RCMH was consistent before the critical point was reached (Fig. 7). Similarly, the time to reach the critical point also did not vary within groups with the same thickness. For example, for the three groups with a thickness of 2 mm, it took 10 min for each group to reach the critical point. Hence, the time to reach the critical point was not affected by the diameter.

However, the RCMH of potato slices of the same thickness but different diameters displayed large differences after the critical point. Both the time to reach the termination point, as well as the RCHM at the termination point was different for each group. For potato slices of the same thickness, the time to reach the termination point positively correlated with the diameter, i.e. it took longer to reach the termination point as the diameter increased. Similarly, the RCMH of potato slices at the termination point also positively correlated with the diameter for potato slices of the same thickness.

To further investigate the change of the RCMH between the critical point and the termination point, the correlation between RCMH and the time value at this stage was modeled. Although the RCMH increased after crossing the critical point threshold, the rising trend gradually tapered with time. Therefore, the trend of the RCMH change between the critical point and the termination point could be captured by the following logarithmic function:

(4) RC=a×ln(t)+b

where *a* and *b* are the parameters to be fitted.

The R2 of each group was greater than 0.9 (i.e. 1 > x > 0.9) (Table 3). This indicated that the variation pattern correlated well with Equation (3). Within groups with the same thickness, parameters *a* and *b* positively correlated with diameter. However, in groups with the same diameter, parameter *a* negatively correlated with thickness. Since equation (3) predicted the RCMH between the critical point and the termination point, the late-stage changes in the potato slice drying process may be predicted with higher accuracy.

**Table 3:** **The logarithmic function fits the data.** The RCMH from the critical point to the termination point.

Thickness (mm)	Diameter (mm)	*a*	*b*	R2^a^	RMSE b (mm)
2	22	74.20	−162.26	0.97	6.02
	33	103.86	−243.07	0.97	9.83
	44	113.86	−269.60	0.91	19.57
4	22	40.92	−163.54	0.98	1.35
	33	72.55	−256.83	0.98	3.40
	44	105.44	−361.85	0.94	9.38
6	22	22.10	−122.64	0.94	1.13
	33	51.33	−242.54	0.97	2.03
	44	66.56	−291.82	0.96	4.63

^a,b^ parameters to be fitted.

a R-squared.

b Root Mean Square Error.

## 4 Conclusions

This paper investigated the deformation of potato slices during hot air drying by building a 3D information acquisition platform of potato slices in the drying process. By converting the collected height information to 2.5D grayscale images, this paper demonstrated the area segmentation and data processing of potato slices using digital image technology. Using this method, the SDMH and RCMH were recorded for each group during the drying process. The time-varying pseudo-color contour map of potato slices showed that the potato slices underwent three stages of regular warping, collapse, and curling during the drying process. After thorough analyses, the parameter RCMH was selected in this study.

By recording the RCMH of potato slices during the drying process, the variation in height was analyzed over time. The drying process reached a critical point at the moment of curling. After that point, the mean height increased and this trend was consistent across different samples. Based on the time-varying characteristics of the mean height, the RCMH and the standard deviation between parallel samples at the critical point and the termination point were extracted and analyzed. The diameter and the slice thickness had little effect on the RCMH and the SDMH at the critical point. At the termination point, both the RCMH and the SDMH increased with increasing diameter. Conversely, as thickness increased, the RCMH and SDMH decreased.

In summary, a proposed functional model could recapitulate the changed pattern of potato slices with different thicknesses and diameters during the critical and termination points. The RCMH at the two points fit this model well. As the coefficient of determination or R2 value of each group was greater than 0.9, the functional model could predict the rate of change of mean height between the critical point and the termination point.

## CRediT authorship contribution statement

**Li Sun:** Data curation, Methodology, Software. **Pengqi Zhang:** Investigation, Writing – original draft. **Xin Zheng:** Software, Validation, Visualization. **Jianrong Cai:** Supervision, Editin. **Junwen Bai:** Writing- Reviewing, Conceptualization.

## Declaration of competing interest

The authors declare that they have no known competing financial interests or personal relationships that could have appeared to influence the work reported in this paper.
